# Aberrant frequency of TNFR2^+^ Treg and related cytokines in patients with CIN and cervical cancer

**DOI:** 10.18632/oncotarget.23581

**Published:** 2017-12-22

**Authors:** Teng Zhang, Jun Jiao, Xinlin Jiao, Lu Zhao, Xinli Tian, Qing Zhang, Daoxin Ma, Baoxia Cui

**Affiliations:** ^1^ Department of Obstetrics and Gynecology, Qilu Hospital, Shandong University, Jinan 250012, Shandong, PR China; ^2^ Department of Obstetrics and Gynecology, Weifang Maternal and Child Health Hospital, Weifang 261011, Shandong, PR China; ^3^ Department of Hematology, Qilu Hospital, Shandong University, Jinan 250012, Shandong, PR China

**Keywords:** cervical cancer, TNFR2, TNFR1, regulatory T cells, cytokines

## Abstract

Regulatory T (Treg) cells expressing tumor necrosis factor receptor 2 (TNFR2) are highly suppressive and are associated with immune homeostasis in various diseases. However, the role of TNFR2^+^Treg subset and relevant cytokines in the development of cervical cancer (CC) remained unclear. In this study, 72 patients with CC, 30 patients with cervical intraepithelial neoplasia (CIN) and 30 healthy volunteers were enrolled. The level of circulating TNFR2^+^Tregs was investigated through flow cytometry. The plasma concentrations of soluble TNFR1 (s-TNFR1) and soluble TNFR2 (s-TNFR2) were determined by enzyme-linked immunosorbent assay. In addition, the mRNA expression levels of TNF-α, TNFR1, TNFR2, and Foxp3 were measured using real-time polymerase chain reaction. Results showed that both peripheral and tumor infiltrating TNFR2^+^Tregs significantly increased in patients with CIN and CC and levels of circulating s-TNFR1 and s-TNFR2 increased in patients with CC. Moreover, the percentage of peripheral TNFR2^+^Tregs was inversely correlated with the clinical stages of CC. Furthermore, the mRNA expression levels of TNF-α, TNFR2, and Foxp3 increased in patients with CIN and CC. Overall, these results indicate that TNFR2^+^Tregs and relevant cytokines contribute to CC development and are promising targets in future immunotherapeutic approaches.

## INTRODUCTION

In 2012, the number of new cases of cervical cancer (CC) was 527,600, whereas the number of deaths due to this disease reached 265,700. CC has become the second most frequently diagnosed tumor and the third leading cause of malignant deaths among women in developing countries [1]. In China, an increasing prevalence of CC was found in young patients [2, 3].

Novel therapeutic strategies to treat CC have undergone significant development, but the overall efficiency of these strategies remains poor. This outcome can be attributed to the capability of tumor cells to escape from the host immune surveillance. Evasion of immune destruction has become a newly-discovered hallmark of cancer [4]. Hence, understanding the mechanism underlying tumor immune escape is important for fabricating novel immunotherapeutic approaches.

During cancer progression, regulatory T (Treg) cells dynamically contribute to establish the immune suppressive condition, which to a large extent hampers anti-tumor immune responses. High Treg cell frequency is closely related to poor prognosis in various tumors, such as breast cancer, renal cell carcinoma, non-small cell lung cancer, and pancreatic ductal cancer. Accumulating evidence suggests that Tregs are made up of heterogeneous subpopulations. Aside from the well-known surface markers CD4 and CD25, biomarkers foxhead box P3 (FoxP3), CD127^low^, CD39, CD73, glycoprotein A repetitions predominant, and cytotoxic T-lymphocyte-associated protein 4 (CTLA-4) also comprise the functional subpopulations of Treg cells [5–8]. Diversity of the Treg markers is associated with the functional characteristics.

Tumor necrosis factor receptor 2(TNFR2), combined with the simultaneous expression of CD4 and CD25, identifies the maximally suppressive subgroups of Tregs in both mice and human beings [9, 10]. Moreover, TNFR2 is involved in the homogeneous expansion of Tregs, rendering it a potential target for manipulating Tregs in the treatment of various diseases [11]. However, to the best of our knowledge, the role of TNFR2^+^Tregs in CC progression remains unclear. In the present study, we examined the level of TNFR2^+^Tregs in both peripheral blood (PB) and tumor infiltrating lymphocytes (TILs), and relevant cytokines in patients with cervical intraepithelial neoplasia (CIN) III and different stages of CC. The relationship between the level of circulating TNFR2^+^Tregs and clinicopathological factors was also investigated. This study aimed to explain the role of TNFR2^+^Tregs in CC development and to provide information for the manipulation of Treg cells in future immunotherapeutics.

## RESULTS

### Circulating TNFR2^+^Tregs showed higher levels in patients with CIN and CC

**Figure 1:**
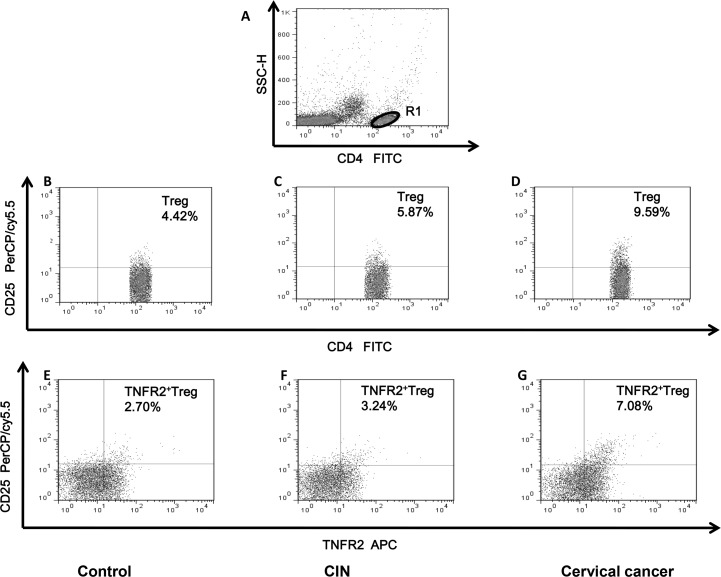
Dot plots of circulating Tregs and TNFR2^+^Tregs in representative patients with CIN, CC and healthy controls **(A)** CD4^+^T cells were gated in R1 by flow cytometry. **(B, C, D)** The proportion of circulating Treg (CD4^+^CD25^+^) cells in healthy controls and patients with CIN and CC. The number in the quadrant represents CD25 expression in the CD4^+^ subsets. **(E, F, G)** Representative dot plots of TNFR2 and CD25 expression in the CD4^+^T cell subsets from healthy controls, CIN and CC patients. The percentage of circulating TNFR2^+^Treg (TNFR2^+^CD25^+^CD4^+^) cells were shown in the upper right quadrant.

We first measured the expression of surface markers CD4, CD25, and TNFR2 on peripheral blood mononuclear cells (PBMCs) to evaluate the percentage of TNFR2^+^Tregs in the peripheral blood of patients with CC, patients with CIN III, and in healthy controls. The population of CD4^+^CD25^+^Tregs as a proportion of total CD4 cells was determined according to the isotype control. Furthermore, the percentage of TNFR2^+^CD25^+^ cells within the gated CD4^+^ T cells was analyzed, and typical dot plots of target cells in representative patients with CC, patients with CINIII, and in healthy controls are shown in Figure 1.

**Figure 2:**
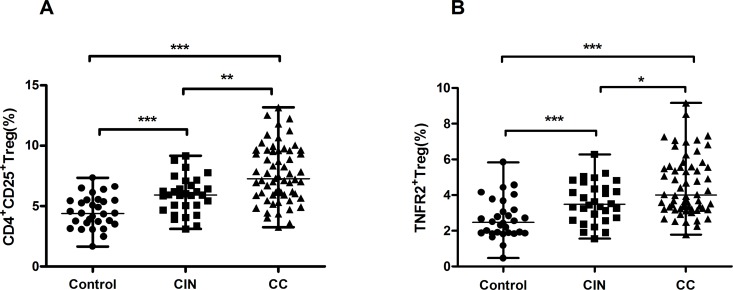
Results of circulating Tregs and TNFR2^+^Tregs in patients with CIN, CC and healthy controls **(A)** The frequency of circulating Treg (CD4^+^CD25^+^) cells in different groups. Compared to healthy controls, there was a markedly higher percentage of Tregs in patients with CC and CIN. Moreover, patients with CC also showed an elevated level of Tregs than patients with CIN. **(B)** The percentage of circulating TNFR2^+^Tregs in different groups. CC and CIN patients showed increased proportion of TNFR2^+^Tregs than healthy controls. Similarly, CC patients displayed an increasing percentage of TNFR2^+^Tregs in comparison with CIN. Data were presented as median, range. *P* values were acquired from Kruskal-Wallis test and Mann-Whitney *U* test. ^*^P<0.05, ^**^P<0.01, ^***^P<0.001.

The percentage of CD4^+^CD25^+^Tregs was markedly higher in the peripheral total CD4 cells of patients with CC (median=7.12%, range, 3.25-13.16%, *P*<0.001) and CIN (median=5.97%, range, 3.11-9.16%, *P*<0.001) than in those of healthy controls (median=4.49%, range, 1.66-7.34%). A similar increasing trend was also detected between patients with CC and patients with CIN (*P*=0.001) (Figure 2A). Furthermore, the proportion of TNFR2^+^Tregs was higher in patients with CC (median=4.07%, range, 1.78-9.16%, *P*<0.001) and CIN (median=3.54%, range, 1.55-6.28%, *P*<0.001) than in healthy controls (median=2.40%, range, 0.47-4.57%). In addition, the level of peripheral TNFR2^+^Tregs was higher in patients with CC compared with that in patients with CIN (*P*=0.013) (Figure 2B).

### Prevalence of up-regulated TNFR2^+^Tregs in tumor infiltrating lymphocytes from patients with CC

**Figure 3:**
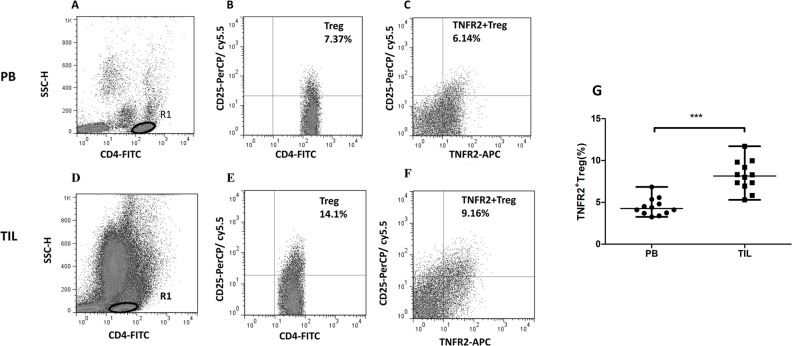
Comparison of TNFR2^+^Tregs between peripheral blood (PB) and tumor infiltrating lymphocytes (TILs) from patients with CC **(A, B, C)** Dot plots of TNFR2^+^Tregs from PB in the representative patient with CC; **(D, E, F)** Dot plots of TNFR2^+^Tregs from TILs in the patient with CC; the same gating scheme mentioned in Figure 1 was used. **(G)** There was a higher proportion of TNFR2^+^Tregs in TILs compared with PB in patients with CC. Data were presented as median, range. *P* values were acquired from Mann-Whitney *U* test. ^***^P<0.001.

We further compared the level of TNFR2^+^Tregs between tumor infiltrating lymphocytes (TILs) and peripheral blood (PB) from 12 patients with CC. A typical flow cytometry result of representative CC patient was shown in Figure 3A-3F. There was a significant increase in the percentage of TNFR2^+^Tregs in TILs (median=8.14%, range, 5.29-11.7%, *n*=12) compared with PB (median=4.25%, range, 3.25-6.84%, *n*=12) (*P*<0.001).(Figure 3G).

### Plasma concentrations of soluble TNFR1(s-TNFR1) and soluble TNFR2(s-TNFR2) increased in patients with CC

**Figure 4:**
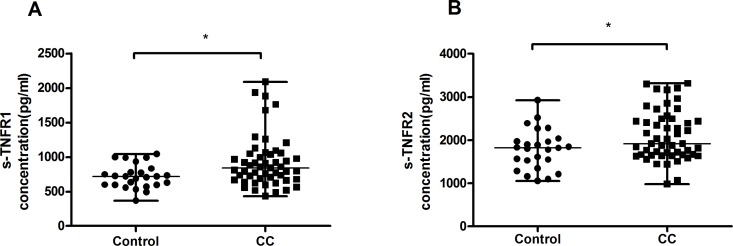
Results of cytokines in plasma from healthy controls and patients with CC **(A)** Concentration of peripheral s-TNFR1 in patients with CC was significantly higher than that of healthy controls. **(B)** There was an increase of plasma s-TNFR2 concentration in patients with CC in comparison with healthy controls. Data were presented as median, range. *P* values were acquired from Mann-Whitney *U* test. ^*^P<0.05, ^**^P<0.01, ^***^P<0.001.

The plasma levels of s-TNFR1 and s-TNFR2 in patients with CC and in healthy controls were determined by enzyme-linked immunosorbent assay (ELISA). As shown in Figure 4A, patients with CC (median=843.4pg/mL; range, 434.9-2087.6pg/mL, *n*=51, *P*=0.011) displayed higher levels of s-TNFR1 compared with healthy controls (median=718.0pg/mL; range, 369.9-1045.3pg/mL, *n*=25). A similar increase was observed in the concentration of s-TNFR2 in patients with CC (median=1917.8pg/mL; range, 983.1-3317.1pg/mL, *n*=51, *P*=0.046) compared with healthy controls (median=1817.2pg/mL; range 1051.2-2924.4pg/mL, *n*=25) (Figure 4B).

### Expression levels of TNF-α, TNFR1, TNFR2, and FoxP3 in patients with CC, patients with CIN, and in healthy controls

**Figure 5:**
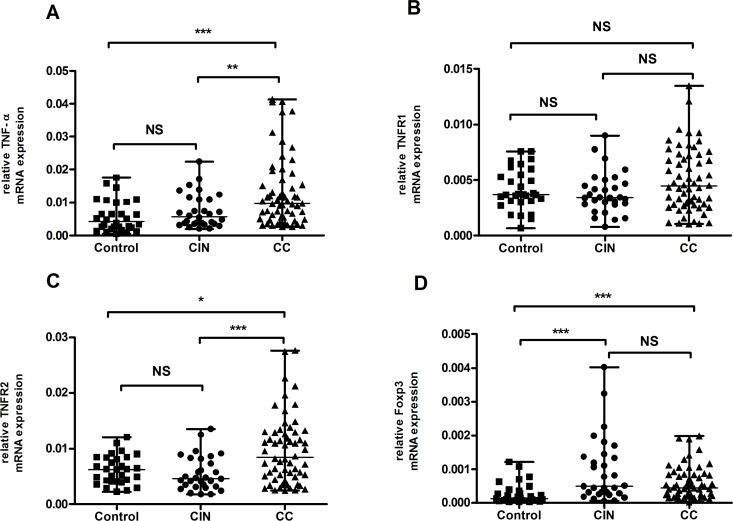
Results of mRNA expression level of TNF-α, TNFR1, TNFR2 and FoxP3 in healthy controls, patients with CIN and CC **(A)** There was a higher expression of TNF-α mRNA in patients with CC in comparison with either patients with CIN or healthy controls. **(B)** No statistic difference in TNFR1 mRNA expression was observed among each group. (P > 0.05). **(C)** Expression level of TNFR2 was significantly increased in patients with CC when compared to patients with CIN and healthy controls respectively. **(D)** Compared to healthy controls, both patients with CC and CIN showed marked elevations in FoxP3 mRNA expression. Data were presented as median, range. *P* values were acquired from Mann-Whitney *U* test. ^**^P<0.01, ^***^P<0.001. NS, no significance.

Patients with CC (median=0.0098, range, 0.0027-0.0413) showed significantly higher mRNA expression levels of tumor necrosis factor α (TNF-α) than patients with CIN (median=0.0057, range, 0.0020-0.0224, *P*=0.009) or healthy controls (median=0.0043, range, 0.0006-0.0176, *P*<0.0001). Nonetheless, no significant difference was found between patients with CIN and healthy controls (*P*>0.05) (Figure 5A).

No statistical difference in TNFR1 expression was observed among patients with CC (median=0.0045, range, 0.0010-0.0135), patients with CIN (median=0.0034, range, 0.0008-0.0090) and healthy controls (median=0.0037, range, 0.0007-0.0076)(*P*>0.05) (Figure 5B). By contrast, the mRNA expression of TNFR2 was significantly higher in patients with CC (median=0.0084, range, 0.0024-0.0276) than in patients with CIN (median=0.0046, range, 0.0018-0.0135, *P*<0.001) and healthy controls (median=0.0063, range, 0.0023-0.0121, *P*=0.010), respectively. However, patients with CIN and healthy controls showed no statistical difference in the mRNA expression of TNFR2 (*P*>0.05) (Figure 5C).

The expression level of Foxp3 was much higher in patients with CC (median=0.00045, range, 0.00003-0.00198, *P*<0.001) and patients with CIN (median=0.00050, range, 0.00006-0.00403, *P*<0.001) compared with healthy controls (median=0.00014, range, 0.00003-0.00122), but no significant difference was found between CC patients and CIN patients (*P*>0.05) (Figure 5D).

### Correlation among circulating TNFR2^+^Tregs, s-TNFR1, s-TNFR2, and clinical characteristics of patients with CC

**Figure 6:**
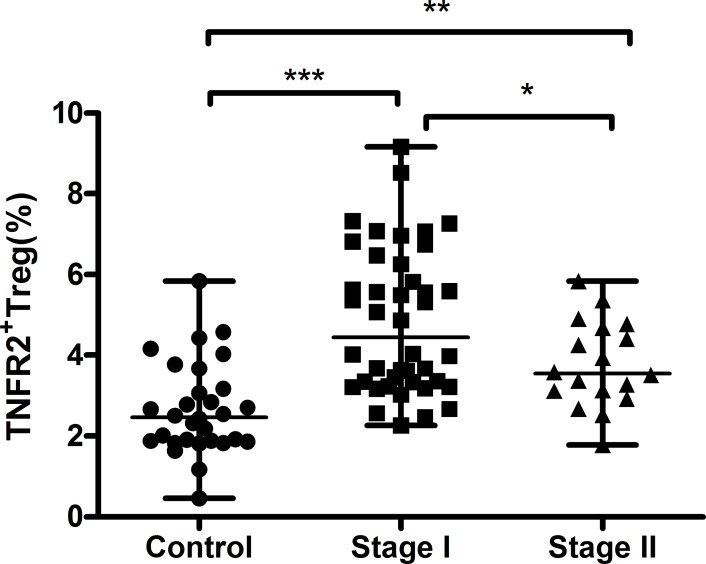
Circulating frequency of TNFR2^+^Tregs from healthy controls, stageI and stageII cervical cancer patients Percentages of peripheral TNFR2^+^Tregs were higher in patients with stageI CC compared to patients with stageII CC. However, patients in both stages displayed profoundly higher proportions of TNFR2^+^Tregs than healthy controls. Data were presented as median, range. *P* values were acquired from Mann-Whitney *U* test. ^*^P<0.05, ^**^P<0.01, ^***^P<0.001.

The peripheral frequency of TNFR2^+^Tregs displayed an inverse relationship with clinical stages, that is, patients with stage I CC (median=4.45%, range, 2.26-9.16%) exhibited a higher percentage of TNFR2^+^Tregs than those with stage II CC (median=3.56%, range, 1.78-5.84%, *P*=0.044) (Figure 6). No statistical differences were discovered between the proportion of TNFR2^+^Tregs and other clinicopathological factors, including lymph node metastasis, tumor size, tumor differentiation, age, and lymphovascular invasion, in patients with CC.

Furthermore, patients with stage II CC showed higher plasma levels of s-TNFR1(median=879.0pg/mL; range, 517.3-1931.4pg/mL) and s-TNFR2(median=1973.7pg/mL; range, 1440.3-3211.2pg/mL) compared with patients with stage I CC (s-TNFR1: median=799.4pg/mL, range, 434.9-2087.6pg/mL; s-TNFR2: median=1919.9pg/mL, range 983.1-3317.1pg/mL), but the difference was not significant (*P*>0.05).

## DISCUSSION

TNFR2 is a transmembrane receptor that can bind to TNF-α, a pleiotropic cytokine involved in regulating the tumor microenvironment [12]. TNFR1 is ubiquitously expressed, whereas TNFR2 is expressed mainly on immune cells and endothelial cells under most physiological circumstances [13]. Transmembrane TNF-α (mTNF) and soluble TNF-α activate TNFR1, whereas TNFR2 is effectively activated mainly by combining with mTNF [14]. TNFR2 preponderantly expressed by Tregs is involved in TNF-α boosted Treg activation, expansion, and homeostasis [15–17]. Contrary to an immunoprotective role in autoimmune pathogenesis, the immunosuppression triggered by Tregs could be detrimental to effective anti-cancer immune responses [18].

In the current study, we examined the frequency of TNFR2^+^Tregs in patients with CC, patients with CIN, and in healthy controls. Our results demonstrated a progressive elevation in the percentage of peripheral TNFR2^+^Tregs in patients with CIN and CC compared with healthy controls, which agreed with previous studies on different types of tumor. Meanwhile, we also detected a dramatic increase in the proportion of TNFR2^+^Tregs in TILs compared to PB, indicating a recruitment of TNFR2^+^Tregs into the tumor microenvironment from the periphery, which possibly contributed to the tumor immune evasion. An elevated level of TNFR2^+^Tregs is correlated with highly immunosuppressive microenvironment in malignant diseases, such as acute myeloid leukemia (AML) [19], lung cancer [20], and ovarian cancer [21], as well as in autoimmune disorders including type 1 diabetes and rheumatoid arthritis [22, 23]. *Ex vivo* experiments also demonstrated a novel regulatory role of TNFR2 on Treg cell function and expansion [24–26]. Govindaraj et al. found that AML patients in remission showed an immunosuppressive status featured by a higher level of TNFR2^+^Tregs, augmenting the propensity for disease relapse [27]. Overall, the incremental levels of TNFR2^+^Tregs might facilitate the tumor progression by fostering an immunosuppressive environment in patients with CC and hampering effective anti-tumor immune responses. The abovementioned aberrant distribution of TNFR2^+^Tregs implies the systemic immunosuppression in CC, which could partly be ascribed to the enhanced suppressive ability of Tregs mediated by a TNF-TNFR2 interaction. Hence, TNFR2 may be a promising target to rectify the immunosuppressive situation mediated by Tregs in patients with CC.

Interestingly, the notably expanded TNFR2^+^Tregs population was inversely correlated with cancer stage. Relative to patients with stage II CC, those with stage I CC displayed a higher percentage of TNFR2^+^Tregs in peripheral blood. Previous studies have shown that chemokines secreted by tumor cells *in situ*, such as CC-motif ligand 22 and CC-motif ligand 28, play an important role in recruiting Tregs into tumor tissues [7, 28, 29]. Thus, we hypothesize that many TNFR2^+^Tregs have undergone trafficking to the *in situ* tumor microenvironment from peripheral circulation as the carcinoma progressed, leading to a decrease in the circulating subsets. This could also be confirmed by our data which showed a much higher level of TNFR2^+^Tregs in TILs compared with PB from the same CC patient.

Such chaos in regulatory T cells may also suggest a losing counterbalance between effector and regulatory T cells in progressive carcinoma. Despite the inhibitory effect of Tregs on effector T (Teff) cells, Teff could boost Treg activation via TNF–TNFR2 interaction [16, 30]. Hence, we hypothesize that the peripheral immunosuppressive environment favors a Treg increment at the initial stage of CC. As the disease progressed, activation and proliferation of Teff were poorly restrained by the expanding Tregs; consequently, Teff performed a depressing ability to boost Treg. Thus, a slightly lower proportion of TNFR2^+^Tregs in stage II was observed in our study. To confirm our hypothesis, further research on the distribution of related effector T cell subsets is needed.

TNFRs also appear in circulating forms, which are mainly generated by shedding from extracellular parts of membrane-bound TNFR by TNF-α-converting enzyme or alternative splicing of receptor transcripts [31]. Soluble TNFRs may act as antagonists for TNF-α or compete with their membrane-anchored counterparts for the ligand, thus neutralizing its proinflammatory and anti-proliferative activities [32, 33]. Conversely, at low concentrations and under certain circumstances, combining with soluble receptors could serve as a mechanism for stabilizing TNF-α and represents a reservoir for the slow-release of TNF-α [34]. Elevated s-TNFR levels and disease progression are correlated in various cases, such as inflammatory bowel disease, colorectal cancer, and chronic kidney disease [35–37]. Accordingly, we observed higher levels of both peripheral s-TNFR1 and s-TNFR2 in patients with CC compared with healthy controls. Enhanced s-TNFR levels possibly provide regulatory effects in response to increasing TNF-α concentration in various solid tumors, including CC.

We further examined the mRNA expression levels of FoxP3, TNF-α, TNFR1, and TNFR2 in PBMCs to determine whether similar changes occur at the transcriptional level in patients with CC. Our results showed that the mRNA levels of FoxP3, TNF-α, and TNFR2 were significantly higher in patients with CC than in healthy controls, whereas TNFR1 did not show a similar trend. However, we could not exclude the possibility for the differential expression of TNFR1, given its ubiquity. Further studies are needed to investigate gene expression in different subsets of CD4^+^ T cells. FoxP3, a major transcription factor for Tregs, plays a vital role in establishing Treg phenotype and promoting their development [38]. In line with a previous report that the expression and shedding of TNFR2 share the same signals [39], upregulated expression of TNFR2 on Tregs and soluble TNFR2 was detected in the present work. Hence, we hypothesize that elevated gene expression is a driving force contributing to the growing population of TNFR2^+^Tregs and soluble TNF-α receptors.

In conclusion, circulating TNFR2^+^Treg, s-TNFR1, and s-TNFR2 were significantly increased in patients with CC. Furthermore, a close correlation was found between TNFR2^+^Treg proportion and clinical cancer stages, suggesting that TNFR2^+^Tregs play a role in CC development. Overall, TNFR2^+^Tregs and relevant cytokines might be associated with CC progression. Thus, TNFR2 may emerge as an attractive target on highly immunosuppressive Treg subsets, which could be tuned by agonists or antagonists to reprogram anti-cancer immune responses for successful cancer immunotherapy. Further studies consisting of *in vitro* and *in vivo* functional assays are needed to elucidate the underlying mechanism.

## MATERIALS AND METHODS

### Patients and healthy volunteers

We enrolled and monitored 72 first-time admitted patients with CC (median age: 47.5 years, range: 26–74 years) and 30 patients with CIN III (median age: 38.5 years, range: 25–50 years) who were pathologically diagnosed at the Department of Obstetrics and Gynecology, Qilu Hospital, Shandong University between September 2014 and September 2017. Participants with autoimmune diseases, a history of any type of malignancies, diabetes, pregnancy, cardiovascular diseases, and active or chronic infections were excluded. The clinical staging of the participants was based on the International Federation of Gynecology and Obstetrics 2009 criteria. Blood samples were collected from all the patients before they received any radiotherapy, chemotherapy, or immunotherapy. Tumor tissue samples were collected from 12 patients with CC during operations. During that period, 30 healthy women (median age: 34.5 years, range: 20–65 years) who received physical examinations in QiluHospital, Shandong University were enrolled as healthy controls. They hold normal results of pap smear (TCT) and HPV tests. Characteristics of the enrolled patients with CC are summarized in Table 1. Informed written consent was obtained from each participant, and the research was conducted in accordance with the Declaration of Helsinki. The Medical Ethical Committee of Qilu Hospital, Shandong University, China provided the ethical approval for the current study.

**Table 1:** Clinical characteristics of cervical cancer patients

Characteristics	Category	N=72 (%)
FIGO stage		
	IA	5 (7%)
	IB	45 (63%)
	IIA	19 (26%)
	IIB	3 (4%)
Histology type	SCC	61 (85%)
	ADC/ADSC	7/2 (9%/3%)
	Others	2 (3%)
Tumor differentiation	Well	10 (14%)
	Moderate	29 (40%)
	Poor	33 (46%)
Lymph node metastases	Positive	17 (24%)
	Negative	55 (76%)
Tumor size (cm)	≤4	53 (74%)
	>4	19 (26%)
Vasoinvasion	Yes	30 (42%)
	No	42 (58%)

Abbreviations: FIGO, International Federation of Gynecology and Obstetrics.

SCC, squamous cell carcinoma; ADC, adenocarcinoma; ADSC, adenosquamous carcinoma.

### Flow cytometric analysis of TNFR2^+^ Treg cells

5 ml of peripheral blood from every subject was collected in heparin-coated tubes before any treatments had done. PBMCs were isolated by Ficoll-Paque combined with density gradient centrifugation. In brief, peripheral blood was diluted with an equal volume of 0.9% saline and mix thoroughly. The diluted blood sample was then carefully layered onto the Ficoll-Paque media solution. After centrifugation at 400×g for 20 min, the mononuclear cell layer at the interface was gently collected and washed twice with 0.9% saline.

Tumor lesions were collected immediately after resection during the surgery and washed with phosphate-buffered saline (PBS) to remove the blood on the surface. Then tissues were cut into 1mm^3^ fragments and incubated with collagenase -IV, hyaluronidase, and DNAse type IV (Sigma) at 37°C for 2 hours. After digestion the mixture was filtrated through a 200 mesh cooper filter to make the single-cell suspension. Tumor infiltrating lymphocytes (TILs) were purified by density gradient centrifugation. The PBMCs and TILs were prepared for flow cytometry using the following procedures.

PBMCs and TILs were re-suspended and incubated at 4°C in the dark for 30 min with a mixture of the following monoclonal antibodies: FITC-conjugated anti-human CD4 (BioLegend, San Diego, CA, USA), PerCP/cy5.5-conjugated anti-human CD25 (BioLegend, San Diego, CA, USA), and APC-conjugated anti-human TNFR2 (R&D Systems Inc., Minneapolis, MN, USA). Simultaneously, isotype controls were established to correct the compensation and to confirm antibody specificity. Then, we washed the mixture and re-suspended the stained cells by adding 300μL of PBS before performing flow cytometry acquisition on a BD FACSCalibur™ flow cytometer. Data were analyzed using Flowjo 7.6.2. Once the CD4^+^lymphocytes were gated, the percentages of Treg (CD4^+^ CD25^+^) and TNFR2^+^ Treg (CD4^+^ CD25^+^TNFR2^+^) in CD4^+^ T cells were further analyzed.

### Determination of s-TNFR levels by ELISA

Immediately after blood was drawn, plasma specimens were obtained by centrifugation and stored at −80°C for further assay. Plasma s-TNFR1 and s-TNFR2 levels were measured by ELISA in accordance with the manufacturer's instructions (R&D Systems Inc., Minneapolis, MN, USA). All samples were examined in duplicate. Standard curves were drawn to calculate the concentrations. The sensitivities of detection were as follows, s-TNFR1, 1.2pg/mL; s-TNFR2, 2.3pg/mL.

### Quantitative real-time polymerase chain reaction analysis

Total RNA was isolated from PBMCs by TRIzol reagent (Takara Bio Inc., China) in accordance with the manufacturer's recommendations. The purity of RNA solution was evaluated by the ratio of the absorbance at 260 and 280 nm using a spectrophotometer (Implen, P330-31). Samples with a ratio between 1.8 and 2.0 were eligible for our study. In total, 1μg of RNA was converted into complementary DNA (cDNA) using a Prime Script RT reagent Kit (Takara Bio Inc., China). Reverse transcription reaction was performed at 37°C for 15 min, followed by 85°C for 5 s. Real-time polymerase chain reaction (PCR) was performed on a Roche Applied Science LightCycler®480II Real-time PCR system (Roche Applied Science, USA) in accordance with the manufacturer's instructions. The PCR reactive system, including 5 μL of 2×SYBR Green Real-time PCR Master Mix, 3.2 μL of ddH_2_O, 1 μL of cDNA, 0.4 μL of the forward primers, and an equal volume of reverse primers, reached a final volume of 10 μL. The primer sequences are shown in Table 2. All samples were examined in triplicate. The PCR products were analyzed by melt curve analysis and agarose gel electrophoresis to estimate product size and to ensure that no by-products were formed. Gene expression was normalized to a housekeeping gene (β-actin) for relative quantification.

**Table 2:** Primers used for quantitative real-time polymerase chain reaction

Gene	Forward primer (5’-3’)	Reverse primer (5’-3’)
TNF-α	CGAGTG ACA AGCCTGTAGC	GGT GTG GGT GAG GAG CAC AT
TNFR1	CCAAGTGCCACAAAGGAACC	CACACCCACAATCAGTCCAA
TNFR2	CAACTCCAGAACCCAGCACT	CACACCCACAATCAGTCCAA
FoxP3	GCTGCAGCTCTCAACGGT	CCTTGAGGGAGAAGACCCCA
β-actin	CCTTCCTGGGCATGGAGTCCTG	GGAGCAATGATCTTGATCTTC

### Statistical analysis

Data distribution was analyzed by Kolmogorov–Smirnov test (K–S test). Results were presented as median (range). Mann–Whitney *U* test and Kruskal–Wallis test were used to assess statistical differences among non-normal distributed data. All tests were performed using SPSS 21 software. Statistical significance was considered at *P*<0.05.

## Abbreviations

CC: cervical cancer
CIN: cervical intraepithelial neoplasia
TNFR1: tumor necrosis factor receptor 1
TNFR2: tumor necrosis factor receptor 2
Treg: regulatory T cells
PBMCs: peripheral blood mononuclear cells
TILs: tumor infiltrating lymphocytes
ELISA: Enzymelinked immunosorbent Assay
PCR: polymerase chain reaction
RNA: ribonucleic acid
mRNA: messenger RNA
